# The Role of Structural Dispersity of Polymer Brushes in Determining the Colloidal Stability of Core–Shell Nanoparticles and Their Interaction with Anti-PEG Antibodies

**DOI:** 10.1021/jacsau.5c00852

**Published:** 2025-08-26

**Authors:** Carlos Pavón, Antonella Grigoletto, Verena Kempkes, Ander Eguskiza, Maria Morbidelli, Roberto Fiammengo, Emanuele Papini, Andrea Mattarei, Gianfranco Pasut, Krzysztof Matyjaszewski, Francesca Lorandi, Edmondo M. Benetti

**Affiliations:** † Laboratory for Macromolecular and Organic Chemistry, Department of Chemical Sciences, 9308 University of Padova, via Marzolo 1, 35131 Padova, Italy; ‡ Department of Pharmaceutical and Pharmacological Sciences, 165490 University of Padova, Via Marzolo 5, 35131 Padova, Italy; § Department of Chemistry, 6612 Carnegie Mellon University, 4400 Fifth Avenue, Pittsburgh, Pennsylvania 15213, United States; ∥ Department of Biotechnology, 19051 University of Verona, 37134 Verona, Italy; ⊥ Department of Biomedical Sciences, University of Padova, 35121 Padova, Italy

**Keywords:** polymer brushes, core−shell nanoparticles, polydispersity, controlled polymerization, surface functionalization

## Abstract

The structural dispersity of poly­[oligo­(ethylene glycol) methacrylate] (POEGMA) brushes critically influences the stabilization of Au nanoparticles (NPs) and their interactions with anti-PEG antibodies (APAs). Commercial oligo­(ethylene glycol) methacrylate (OEG_*p*_MA) macromonomers are intrinsically polydisperse, featuring a distribution of ethylene glycol (EG) units spanning from *n* = 2 to *n* = 15. Flash chromatography was applied to isolate OEG_*8*_MA with discrete length (*n* = 8)—the most abundant species in commercial mixtures. Controlled radical polymerization of polydisperse and discrete monomer sources was subsequently applied to generate POEG_*p*_MA and POEG_*8*_MA, which feature heterogeneous and homogeneous structures, respectively, while displaying an overall identical composition. When used to form shells on Au NPs, uniform POEG_*8*_MA brushes provided enhanced colloidal stability across a wide temperature range compared to their polydisperse counterparts. While serum protein corona formation was largely determined by polymer composition, APA binding was promoted by longer OEG segments present in polydisperse POEG_*p*_MA shells, which acted as epitopes for antibody recognition. These findings highlight how controlling polymer architecture and dispersity in the design of PEG-based shells for NPs could give access to nonimmunogenic formulations. More broadly, polymer dispersity emerges as an additional tool for modulating the behavior of nanomaterials within biological systems.

## Graphical abstract

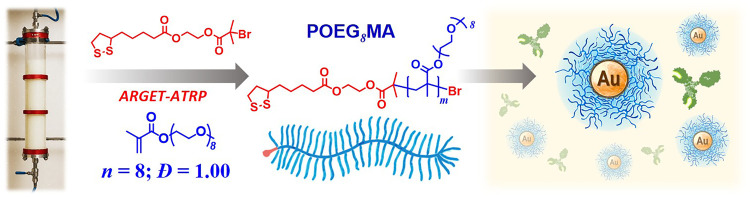

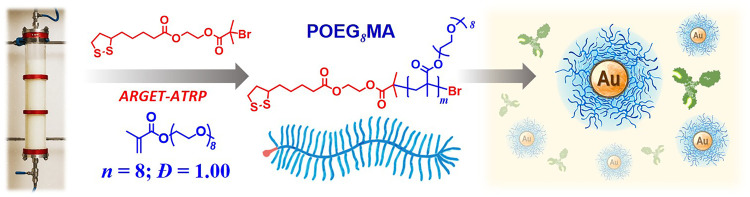

## Introduction

Polymer brush-based shells are commonly applied on nanoparticles (NPs) to ensure their stabilization within physiological environments, and to provide desirable pharmacokinetics by prolonging their blood circulation times.^1–5^ Poly­(ethylene glycol)­s (PEGs) have traditionally been the material of choice for generating hydrated shells that stabilize NPs and provide to them stealth properties within protein-rich media. However, serious concerns over the immunogenicity of PEGylated nanotherapeutics are on the rise and have stimulated researchers to explore alternative solutions.^6,7^

In particular, the application of PEGylated therapeutics can elicit varying levels of anti-PEG antibodies (APAs), potentially compromising the clinical effectiveness of treatments^8,9^ and causing severe immune reactions to patients.^6,7,10^ Recent studies have shown that SARS-CoV-2 mRNA vaccines based on PEGylated lipid nanoparticles (LNPs) contributed to boost APA generation in a significant fraction of individuals.^11^ In addition, up to 70% of people who have never received PEGylated therapies were found to present pre-existing APAs presumably due to the widespread exposure to PEG-based additives present in food and cosmetics.^11,12^

While immunogenicity may pose a serious threat to the efficacy of NP formulations comprising common, linear PEGs, alternative stealth polymers have been emerging. These included poly­(2-oxazolines),^13,14^ polypeptoids,^15^ and polyphosphoesters,^16^ which feature biocompatibility, tunable properties, and reduced immunogenicity compared to PEGs. In parallel, polymers with similar composition but different architecture have been emerging as alternatives—especially in the development of polymer conjugates of protein-based drugs and RNA therapeutics.^17–19^ These are based on poly­[oligo­(ethylene glycol) methacrylate]­s (POEG_*p*_MAs), which feature a graft polymer structure where oligo­(ethylene glycol) (OEG) segments extend from a polymethacrylate backbone.^20,21^ The application of POEG_*2*_MA and POEG_*3*_MA—respectively synthesized from di­(ethylene glycol) methacrylate (OEG_*2*_MA) and tri­(ethylene glycol) methacrylate (OEG_*3*_MA)—in place of linear PEG provided drug conjugates with minimal or no reactivity toward APAs, due to the absence of relatively long OEG sequences that represent epitopes for antibodies.^22^ However, POEG_*2*_MA and POEG_*3*_MA feature lower critical solution temperatures (LCSTs) in aqueous media below or near the body temperature^23–26^ and they form relatively amphiphilic brushes on solid surfaces.^27,28^ Hence, these are not the best candidates for generating highly hydrated brush shells that colloidally stabilize NPs across a broad range of temperatures and quantitatively suppress hydrophobic interactions.

In contrast, POEG_*p*_MAs with *n* > 4 feature longer OEG side chains, are more hydrated in water and show relatively high values of LCST (comparable to those displayed by linear PEGs). In a similar way to PEGs, such POEG_*p*_MAs represent excellent ligands for the robust physicochemical stabilization of NPs within physiological environment.^21,29,30^

However, the application of POEGMA brushes featuring longer OEG segments for the stabilization of NPs within physiological environment presents several drawbacks.

Although providing superior bioinertness toward serum proteins, POEG_*p*_MA brushes from commercial macromonomers were also reported to stimulate reactivity toward APAs, thus making them as immunogenic as PEG-based analogues.^20,22^ Most importantly, we recently highlighted that POEG_*p*_MAs feature a heterogeneous graft-polymer structure, which significantly altered the physicochemical properties of the derived brushes.^31^ In particular, OEG_*p*_MAs (typically featuring *M*_n_ ∼ 300 or ∼500 Da) are intrinsically polydisperse, showing a distribution of *n* included between 2 and 15 (Figure 1). The consequent structural heterogeneity of POEG_*p*_MA brushes favored hydrophobic interactions and reduced hydration of brush films grafted on macroscopic surfaces.^31^

**Figure 1:**
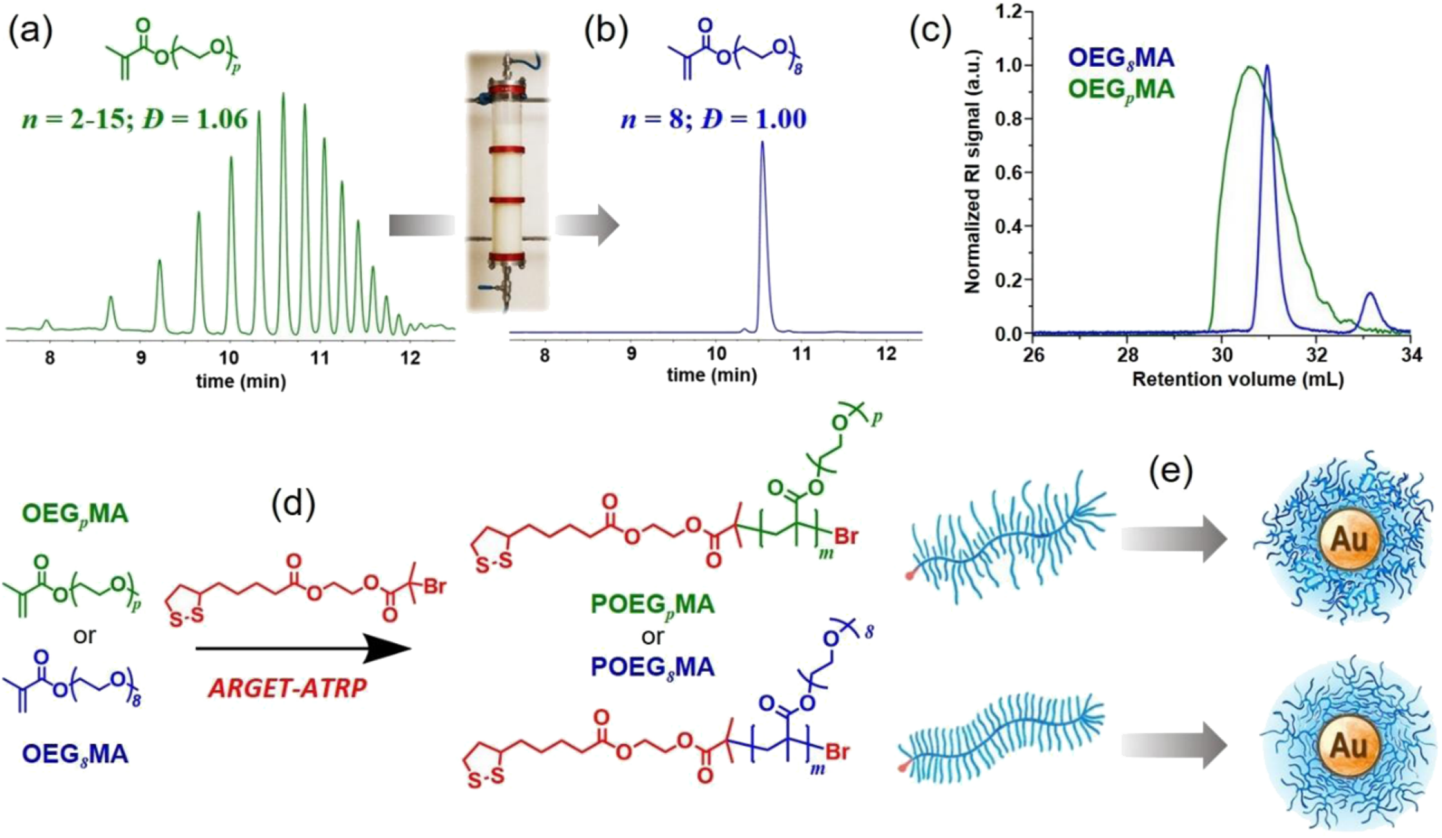
(a) UPLC elugrams displaying the *M*_n_ distribution of OEG_*p*_MA including species from *n* = 2 to *n* = 15 and (b) discrete OEG_*8*_MA with *n* = 8 obtained after chromatographic purification. (c) SEC elugrams of commercial (polydisperse) OEG_*p*_MA and discrete OEG_*8*_MA. (d) ARGET-ATRP from disulfide-based initiators provided structurally heterogeneous POEG_*p*_MA (*M*_n_ = 12.9 kDa, *Đ* = 1.12) and POEG_*8*_MA with homogeneous structure (*M*_n_ = 13.1 kDa, *Đ* = 1.14). (e) POEG_*p*_MA and POEG_*8*_MA species were applied to generate core-polymer brush shell Au NPs (alternatively POEG_*p*_MA@AuNPs and POEG_*8*_MA@AuNPs). Elugrams reported in (a) and (b) were reproduced from ref 31. Copyright 2024 American Chemical Society.

Despite recent advances in dissecting the impact of polymer dispersity on the properties of polymer brushes,^32–38^ the effects of brush’ structural heterogeneity on the behavior of core-polymer shell NPs within media that are relevant for biomedical applications remain unexplored.

While focusing on Au NPs, here we demonstrate that brush shells constituted by structurally homogeneous POEGMAs provide enhanced colloidal stabilization and significantly hinder APA binding when compared to POEG_*p*_MA counterparts that are chemically identical, although structurally polydisperse (Figure 1). In addition, we prove that immunogenicity toward PEG can be largely reduced by controlling the degree of structural heterogeneity of polymer ligands forming shells on NPs, as polydispersity emerges as a parameter directly correlated to the exposure of OEG segments that function as epitopes for antibody-binding.

## Results and Discussion

The distribution of *n* within commercial mixtures of OEG_*p*_MA (with *M*_n_ ∼ 500 Da) is highlighted by ultra performance liquid chromatography (UPLC) coupled with electrospray ionization (ESI) mass spectrometry (Figure 1a,b). Purification of OEG_*p*_MAs through flash chromatography gave access to OEG_8_MA with *n* = 8 (*M*_n_ = 452 Da) as the most abundant species, accounting for ∼15 mol % of the commercial OEG_*p*_MA mixture of macromonomers (Table 1). Interestingly, the observed distribution of macromonomer species in OEG_*p*_MA corresponded to a dispersity (*Đ* = *M*_w_/*M*_n_) of 1.06, whereas the number-average value of molar mass was 466 Da with a standard deviation of ± 117 Da—which gives a more realistic perception of the degree of heterogeneity in macromonomers’ length.^39^

**Table 1:** Structural Properties of POEG_*p*_MA@AuNPs and POEG_*8*_MA@AuNPs

sample	*D*_H_ (DLS) [nm]	PDI^a^	Z-potential [mV]	weight loss (TGA) [%]	*σ* ^b^ [nm^–2^]
citrate@AuNPs	14.3 ± 0.1	0.046 ± 0.009	–30.3 ± 0.6		
POEG_*p*_MA@AuNPs	29.2 ± 0.8	0.090 ± 0.012	–5.3 ± 2.1	11 ± 3	0.26 ± 0.09
POEG_*8*_MA@AuNPs	31.1 ± 0.6	0.099 ± 0.021	–8.5 ± 0.6	15 ± 3	0.32 ± 0.08

a PDI was calculated as PDI = (standard deviation/mean)^2^, the mean and standard deviation were determined based on multiple size measurements reported in the first column of the table.

b σ was calculated as σ = (*n*_ligand_ *N*_A_)/(*N*_NPs_ *A*_NP_), where *n*_ligand_ refers to mols of the polymer, *N*_A_ Avogadro’s number, *N*_NPs_ number of nanoparticles, and *A*_NP_ area of a NP.

Subsequent activator regenerated by electron transfer atom transfer radical polymerization (ARGET-ATRP)^40,41^ of polydisperse OEG_*p*_MA and discrete OEG_*8*_MA from disulfide-bearing initiators alternatively provided structurally heterogeneous POEG_*p*_MA (*M*_n_ = 12.9 kDa, *Đ* = 1.12) and POEG_*8*_MA with homogeneous structure (*M*_n_ = 13.1 kDa, *Đ* = 1.14) with comparable molar mass (Figure 1c). POEG_*p*_MA and POEG_*8*_MA were subsequently applied as ligands to form brush shells on Au NPs, exploiting ligand exchange on freshly synthesized, citrate-functionalized Au NPs (citrate@AuNPs) presenting hydrodynamic diameter (*D*_H_) of 14.3 ± 0.1 nm (Table 1), as measured by dynamic light scattering (DLS). The formation of polymer brush shells was mirrored by an increment in the values of *D*_H_, which were 29.2 ± 0.8 nm and 31.1 ± 0.6 nm, for Au NPs functionalized with POEG_*p*_MA (POEG_*p*_MA@AuNPs) and POEG_*8*_MA (POEG_*8*_MA@AuNPs), respectively. In addition, thermogravimetric analysis (TGA) confirmed the presence of polymer brush layers on Au cores and provided the corresponding values of grafting density (σ), which were 0.26 ± 0.09 and 0.32 ± 0.08 nm^–2^, for POEG_*p*_MA@AuNPs and POEG_*8*_MA@AuNPs, respectively (Table 1).

The presence of dense POEG_*p*_MA and POEG_*8*_MA brushes provided colloidal dispersions with largely increased stability compared to citrate-functionalized Au NPs (citrate@AuNPs) as expected. This was highlighted by both UV–vis spectroscopy and DLS, subjecting suspensions of NPs to aqueous media presenting increasing ionic strengths—generated by addition of NaCl from 0.4 to 2.0 M—and by dispersing them in phosphate buffer saline (PBS) solution (pH = 7.4). Already within 0.4 M NaCl solutions, dispersions of citrate@AuNPs showed aggregation, as witnessed by the appearance of absorption bands at wavelengths >700 nm in the corresponding UV–vis spectra, which were due to dipole coupling between the plasmons of neighboring NPs forming aggregates (Figure 2a).^42–45^ In contrast, POEG_*p*_MA@AuNPs and POEG_*8*_MA@AuNPs formed stable dispersions within the entire range of concentrations of NaCl tested, with UV–vis profiles displaying a single plasmon band centered at λ = 525 nm (Figure 2b,c). Au NPs functionalized with POEG_*p*_MA and POEG_*8*_MA brush shells also showed no sign of aggregation when dispersed in PBS at ambient temperature following incubation for several days (Figure S8).

**Figure 2:**
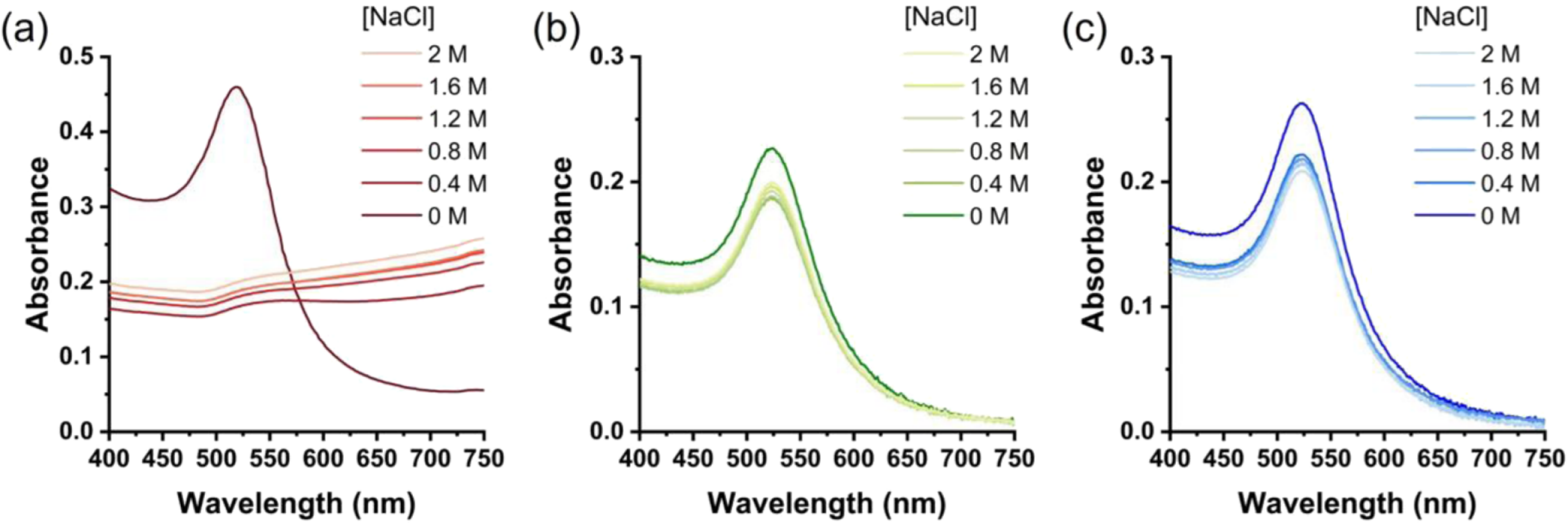
UV–vis spectra recorded for core–shell Au NP suspensions with different concentrations of NaCl after 30 min incubation (a) citrate@AuNPs, (b) POEG_*p*_MA@AuNPs, and (c) POEG_*8*_MA@AuNPs.

The influence of brush structural heterogeneity on colloidal stabilization was highlighted by increasing the temperature of NPs’ suspensions above the LCST of POEG_*p*_MA and POEG_*8*_MA brush shells—thus forcing Au core-brush shell NPs to aggregate—and subsequently monitoring the reversibility of the temperature-induced aggregation process by cooling down the suspensions. This test was proven particularly effective to emphasize the contribution of hydrophobic interactions within polymer brush shells and between shells on different NPs, and to highlight how core–core interactions between NPs are efficiently screened through brush functionalization.^46–48^

Suspensions of 3.5 nM POEG_*p*_MA@AuNPs and POEG_*8*_MA@AuNPs in 2 M NaCl were subjected to gradual heating from 37° to 65 °C while monitoring transmittance of the mixture and the corresponding *D*_H_ of NPs by DLS (Figure 3a,b).

**Figure 3:**
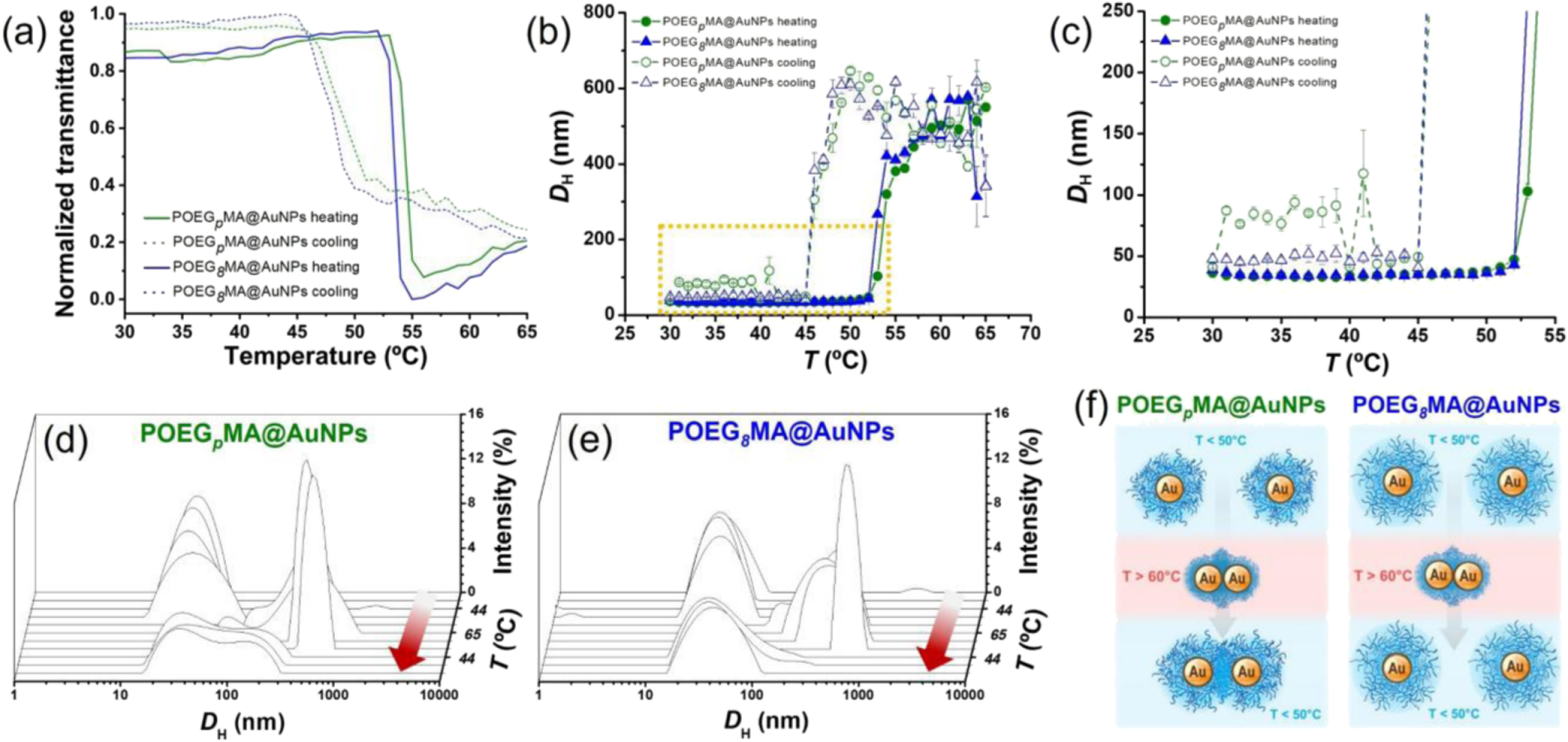
(a) Transmittance profiles recorded at λ = 658 nm by DLS on 3.5 nM dispersions of POEG_*p*_MA@AuNPs and POEG_*8*_MA@AuNPs in 2 M NaCl while increasing temperature from 37 to 65 °C (continuous lines) and cooling down from 65 to 37 °C (dashed profiles) with a heating/cooling rate of 1 °C min^–1^. Three replicates using three different batches of core–shell NPs were used for estimating the corresponding LCST values. (b, c) Intensity-weighted *D*_H_ values recorded by DLS while heating 3.5 nM dispersions of POEG_*p*_MA@AuNPs and POEG_*8*_MA@AuNPs in 2 M NaCl from 37 to 65 °C (filled markers), at a heating rate of 1 °C min^–1^, and while cooling down the same dispersions back to 37 °C at a cooling rate of 1 °C min^–1^ (empty markers). (d) DLS profiles recorded on 3.5 nM POEG_*p*_MA@AuNPs dispersions during a heating–cooling cycle. (e) DLS profiles recorded on 3.5 nM POEG_*8*_MA@AuNPs dispersions during a heating–cooling cycle. (f) Schematics depicting the partially irreversible aggregation of POEG_*p*_MA@AuNPs and the reversible aggregation of POEG_*8*_MA@AuNPs during heating/cooling cycles.

The LCST values of polymer brush shells—corresponding to the temperature at which a 50% drop in transmittance was recorded—are >90 °C for both POEG_*p*_MA and POEG_*8*_MA in ultrapure water.^49^ However, in 2 M NaCl suspensions, LCSTs were recorded in the range 53.5–54.5 °C, showing ∼5 °C of hysteresis during the subsequent cooling ramps and displaying no significant differences when comparing polydisperse and homogeneous brush shells (Figure 3a).

Interestingly, at temperatures above the LCST, the transmittance of both POEG_*p*_MA@AuNPs and POEG_*8*_MA@AuNPs dispersions showed a further increase upon heating. This behavior was due to a partial precipitation of NP aggregates at the bottom of the cuvettes, which were withdrawn from the dispersions and led to a progressive increment of transmittance when temperature was raised above 55 °C.^50^

Analysis of the hydrodynamic size of core-brush shell NPs highlighted the influence of brush dispersity on the stability of NPs during heating/cooling tests. At 37 °C, POEG_*p*_MA@AuNPs and POEG_*8*_MA@AuNPs showed *D*_H_ = 33.0 ± 0.4 and 33.6 ± 1.4 nm, respectively (Figure 3b,c), in both cases with a monomodal distribution (Figure 3d,e). Upon reaching the LCST both suspensions aggregated showing a sharp increment of *D*_H_ (Figure 3b) and the appearance of multiple size distributions with *D*_H_ > 200 nm, which are characteristic clustered NPs (Figure 3d,e).

Subsequent cooling of the suspensions to the starting temperature of 37 °C led to different DLS profiles by comparing POEG_*p*_MA@AuNPs and POEG_*8*_MA@AuNPs. In the latter case, NPs disaggregated and showed monomodal DLS profiles, like those displayed by the same suspensions before the heating ramp (Figure 3e). In contrast, POEG_*p*_MA@AuNPs did not recover the initial size distribution and maintained relatively broad and multiple DLS signals, indicating that the aggregation process was partially irreversible (Figure 3d). In other words, NPs presenting structurally heterogeneous POEG_*p*_MA brushes once forming aggregates above LCST could not be quantitatively redispersed by lowering the temperature (Figure 3f). This phenomenon was likely due to hydrophobic interactions between POEG_*p*_MA shells, which arose when these are forced to interact within NPs’ aggregates,^31^ presumably coupled to an inefficient screening of core–core interactions that emerged when different NPs form tightly associated aggregates above LCST.^46^ In contrast, Au NPs presenting POEG_*8*_MA brushes with homogeneous structure can be readily redispersed upon cooling below LCST as a result of improved hydration and the formation of a more uniform shell that effectively shields interactions between the Au cores (Figure 3f).

The reduced steric stabilization and the occurrence of hydrophobic interactions between POEG_*p*_MA brush shells within aggregates of NPs could also be confirmed indirectly through atomic force microscopy (AFM) measurements (Figure 4). POEGMA brushes were grafted onto 1.5 × 1.5 cm^2^ Au substrates exhibiting dry thickness (*T*_dry_) measured by variable angle spectroscopy ellipsometry (VASE) of 2.4 ± 0.5 and 2.3 ± 0.3 nm for POEG_*p*_MA and POEG_*8*_MA, respectively.

**Figure 4:**
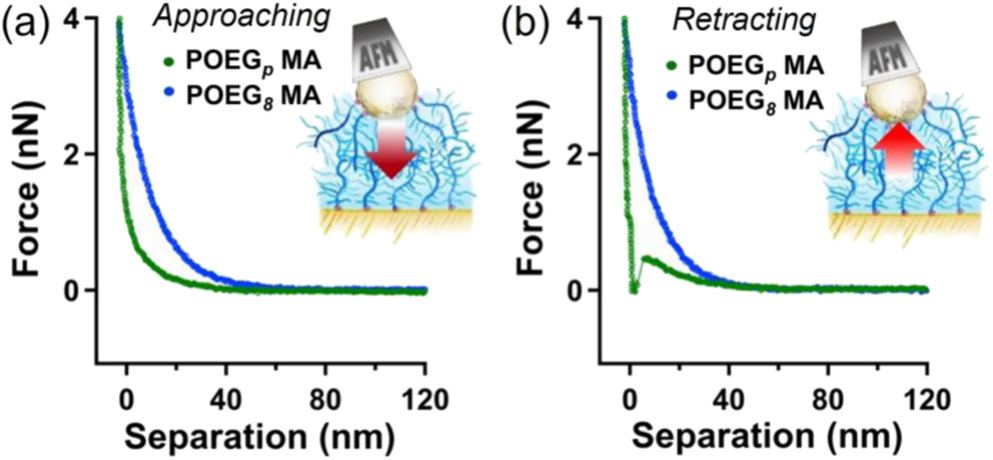
FS curves recorded on POEG_*p*_MA and POEG_*8*_MA brushes deposited on macroscopic Au surfaces. In (a) approaching FS profiles of POEG_*p*_MA (green traces) and POEG_*8*_MA brushes (blue traces) are reported. In (b) the corresponding retracting FS profiles for POEG_*p*_MA (green traces) and POEG_*8*_MA brushes (blue traces) are displayed.

When POEG_*p*_MA brushes assembled on an Au-coated, 20 μm colloidal AFM probe were compressed against an identical brush countersurface, a lower steric stabilization was recorded with respect to that generated between structurally homogeneous POEG_*8*_MA brush counterparts. This was evidenced by comparing the approaching profiles of force–separation (FS) curves recorded while compressing the two different brush types (Figure 4a), where a higher contribution of repulsive forces clearly characterized structurally homogeneous POEG_*8*_MA brushes under mechanical compression.

In addition, the comparative analysis of retracting FS profiles highlighted how polydisperse POEG_*p*_MA brushes compressed on identical assemblies generated adhesive interactions, whereas for POEG_*8*_MA analogues retracting profiles nearly overlapped approaching curves witnessing no sign of adhesion (Figure 4b).

The different stabilizing properties displayed by POEG_*p*_MA and POEG_*8*_MA brushes did not translate into a significantly different interaction with serum proteins by the corresponding core–shell Au NPs. When POEG_*p*_MA@AuNPs and POEG_*8*_MA@AuNPs were incubated in a 1 mg mL^–1^ bovine serum albumin (BSA) solution in PBS buffer (pH = 7.4) no signs of aggregation phenomena were recorded in both cases, with DLS profiles showing analogous size distributions compared to those characteristics of protein-free media (Figure 5a). A similar behavior was also confirmed by UV–vis spectroscopy (Figure S9).

**Figure 5:**
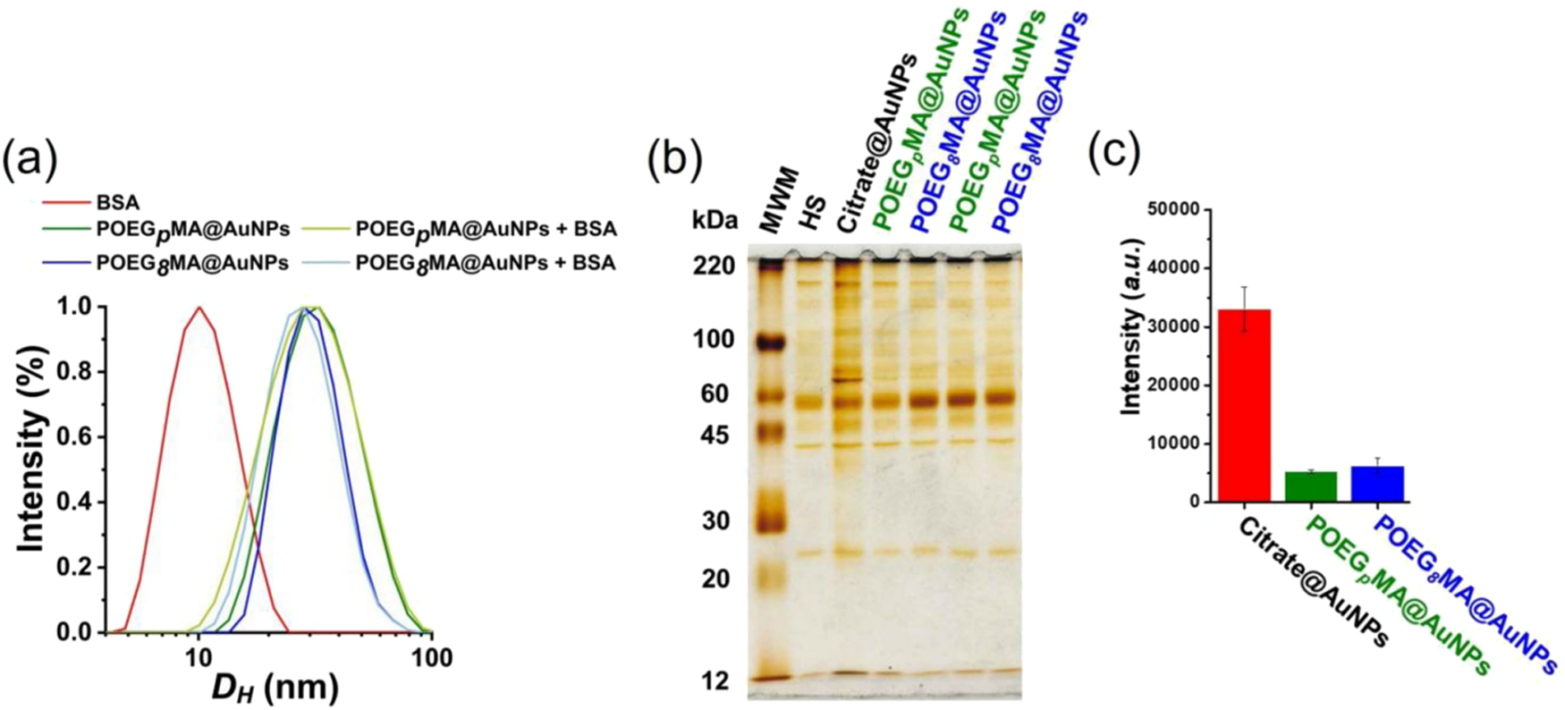
(a) DLS profiles displaying normalized intensity-weighted *D*_H_ of 3.5 nM citrate@AuNPs, POEG_*p*_MA@AuNPs, and POEG_*8*_MA@AuNPs before (darker colors) and after (light green and blue, respectively) addition of 1 mg mL^–1^ BSA. (b) SDS-PAGE of 2.5 nM citrate@AuNPs, POEG_*p*_MA@AuNPs, and POEG_*8*_MA@AuNPs. The first lane shows the protein bands corresponding to the molecular weight marker (MWM), used as a reference to identify protein sizes. The second lane (HS) is a control displaying signals from residual proteins that remained at the bottom of the Eppendorf without the addition of NPs. (c) Histograms present a semiquantitative estimation of the proteins associated with NPs, obtained by densitometric analysis of the corresponding SDS-PAGE bands after silver staining. Bands intensities were quantified using ImageJ software, following subtraction of background signals (*i.e.*, HS). Histograms show relative intensity of two replicates for every type of NP.

A certain interaction with serum proteins was anyhow unavoidable and it could be evaluated by subjecting core–shell NPs to 60% human serum (HS) (Figure 5b,c) even for a relatively short, 20 min incubation at 37 °C. This protocol—validated in previous studies^51,52^ —evidenced the most abundant or stronger binding serum proteins, which likely reach an association equilibrium with NPs already within a few minutes (Figure 5b,c). The major proteins bound to citrate@AuNPs, POEG_*p*_MA@AuNPs and POEG_*8*_MA@AuNPs were subsequently analyzed by sodium dodecyl sulfate-polyacrylamide gel electrophoresis (SDS-PAGE). Silver staining highlighted the bands referring to different proteins interacting with NPs as a function of their apparent molar mass (Figure 5b). Subsequent densitometry on the protein bands, followed by subtraction of the signals generated by the residual unbound proteins—which remained in the samples while not being associated to NPs—provided a semiquantitative estimation of the amount of proteins forming coronas on each type of NPs (Figure 5c).^53,54^

Overall, the amount of serum proteins associated to NPs was significantly suppressed when these were functionalized with POEG_*p*_MA and POEG_*8*_MA brushes compared to citrate@AuNPs in agreement with the stealth properties of POEGMAs. However, under these conditions, no significant differences were found between polydisperse and structurally homogeneous brushes. Hence, structural dispersity of brushes did not lead to a substantial variation in the interaction of NPs with serum proteins.

Whereas POEG_*p*_MA and POEG_*8*_MA brushes interacted in a comparable way with serum proteins, their different structural properties led to a significant shift in the response toward APAs.

This was demonstrated by surface plasmon resonance (SPR) spectroscopy, investigating the intrinsic tendency of POEG_*p*_MA and POEG_*8*_MA brushes assembled on Au surfaces to bind APAs.^55^ We specifically focused on the interaction of monodisperse and polydisperse brush shells with monoclonal APAs that are “methoxy-specific”, *i.e.*, which bind to the terminal methoxy moieties of PEG segments including up-to seven ethylene glycol (EG) repeating units.^56^ Au-coated SPR sensors functionalized with brushes were initially subjected to 0.15 μM solution of BSA, to rule out any contribution from unspecific adsorption of APAs on the polymer interface. Later, solutions with increasing concentration of APAs were injected into the sensor, while recording wavelength shift as a function of incubation time (Figure 6a,b).

**Figure 6:**
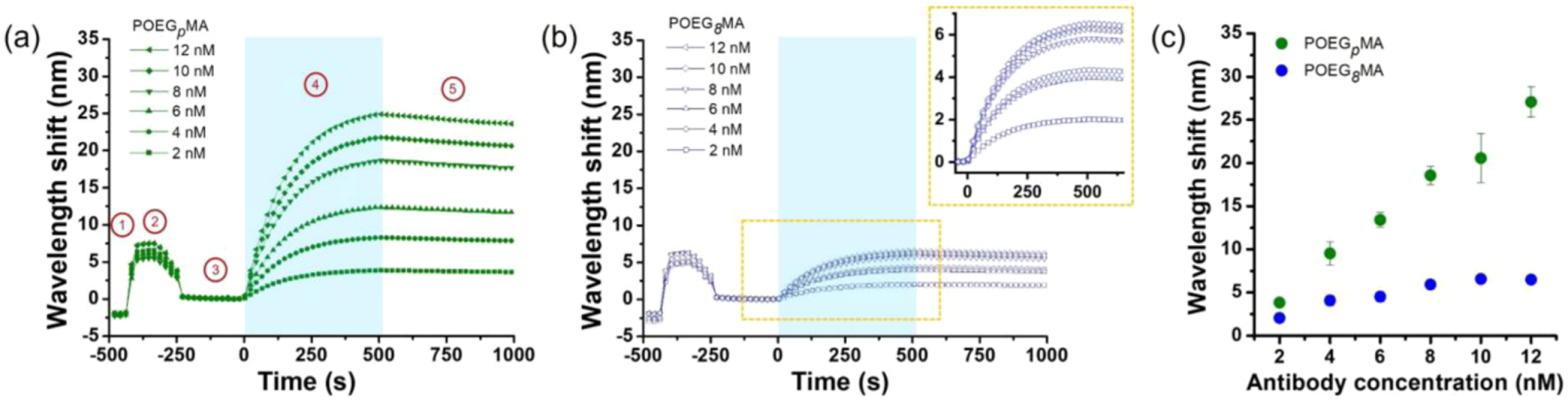
SPR sensograms obtained by subjecting POEG_*p*_MA (a) and POEG_*8*_MA brushes (b) assembled on Au SPR chips to solutions with increasing concentration of APAs (2–12 nM). Brush-functionalized SPR chips were incubated in HEPES buffer (1) followed by a 0.15 μM solution of BSA (2). After 180 s of incubation, polymer brush assemblies were rinsed with HEPES buffer (3). At time 0, the solution of APA was injected (4), and after 540 s (association step), HEPES buffer was injected (5, dissociation step) for an incubation of 900 s. (c) Wavelength shift vs APA concentration obtained from the SPR data for APA binding to a surface grafted with POEG_*p*_MA and POEG_*8*_MA brushes.

APAs showed very similar binding profiles while comparing polydisperse POEG_*p*_MA and monodisperse POEG_*8*_MA assemblies (Figure 6a and inset in Figure 6b), suggesting an analogous kinetics of interaction on structurally different POEGMA brushes. However, for each APA concentration tested much lower values of wavelength shifts were recorded on homogeneous POEG_*8*_MA films if compared to POEG_*p*_MA counterparts (Figure 6c).

On structurally heterogeneous POEG_*p*_MA brushes an increment of APA concentration between 2 and 12 nmol L^–1^ was mirrored by a concomitant increase in wavelength shift, which was nearly linear and indicated significant, concentration-dependent binding of antibodies. In contrast, POEG_*8*_MA brushes with homogeneous structure showed much lower interaction with APAs. A relatively low binding was recorded until APA concentration reached 4 nmol L^–1^ and surface saturation was observed above 8 nmol L^–1^ (Figure 6c).

The significantly lower binding of APAs on POEG_*8*_MA brushes was also recorded by surface fluoroimmunoassay (Figure S10), confirming the reduced reactivity of structurally monodisperse grafts.

These results indicated that, although brush dispersity did not alter the mechanism of APA interaction, monodisperse POEG_*8*_MA brushes triggered binding with much fewer antibodies with respect to their structurally heterogeneous analogues. Relevantly, at the highest APA concentration tested of 12 nM, a nearly 5-fold higher wavelength shift was recorded on POEG_*p*_MA brushes compared to the corresponding POEG_*8*_MA, which correlated to a proportionally higher mass of APAs.

The application of POEGMAs in place of linear PEGs was already reported to alter the binding of methoxy-specific APAs on different types of biomaterials. The team of Chilkoti demonstrated that reducing the length of OEG_*n*_ side chains to *n* = 3 prevented interaction with APAs both on brush coatings and on POEGMA bioconjugates.^17–20^ More recently, Siegwart and co-workers demonstrated that APA binding on POEG_*p*_MA grafts forming shells on lipid nanoparticles (LNPs) was significantly influenced by the density of grafts and their overall molar mass (*i.e*., backbone length)^57^—both these factors determining the conformation of POEG_*p*_MAs at the interface of LNPs.

Here we have demonstrated that structural dispersity of POEGMA brushes emerges as an additional parameter determining APA binding. Commercial macromonomer mixtures feature more than 60 mol % of OEG_*n*_MAs with *n* ≥ 8, virtually generating brushes with a high concentration of dangling OEG segments that extend from the surface functioning as epitopes for antibody binding (Figure 7a). In contrast, structurally homogeneous POEG_8_MA brushes form a morphologically uniform layer, with a much lower content of OEG segments available at the interface for APA binding (Figure 7b).

**Figure 7:**
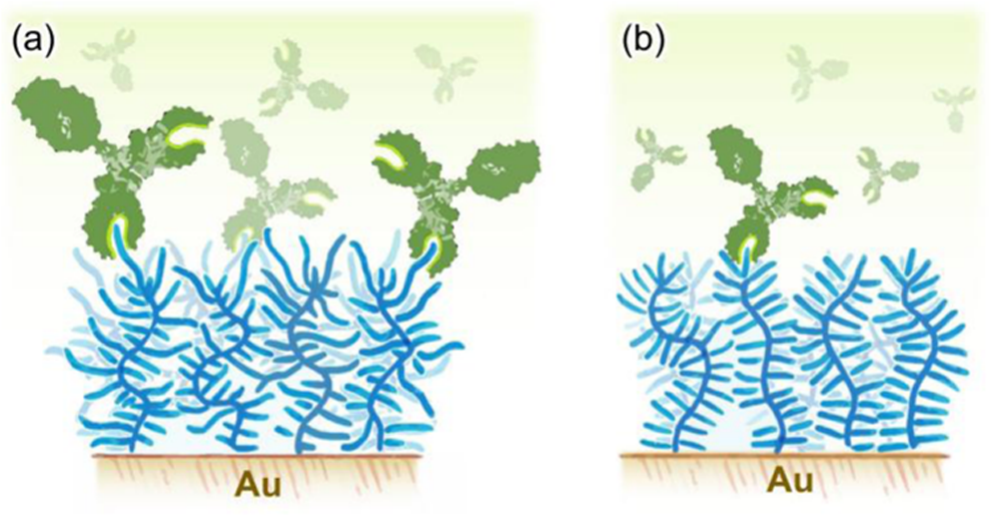
Schematics depicting the interaction between APAs and structurally different POEGMA brushes. (a) Polydisperse POEG_*p*_MA brushes present a high concentration of dangling OEG segments at the interfaces, which function as epitopes for APA binding. (b) POEG_*8*_MA brushes form more uniform layers that suppress interaction with antibodies.

## Conclusions

In this work, we highlighted how the structural dispersity of POEGMA brushes emerges as a determinant parameter for the stabilization of Au NPs and for their interaction with APAs. Purification of polydisperse OEG_*p*_MAs through flash chromatography enabled us to isolate discrete OEG_*8*_MA, which is the most abundant species present in the commercial macromonomer mixtures. Subsequent controlled radical polymerization was employed to generate structurally polydisperse POEG_*p*_MA and the corresponding POEG_*8*_MA, which present an overall identical composition but a homogeneous structure.

When applied to form shells on Au NPs, structurally homogeneous POEG_*8*_MA brushes provided an increased colloidal stability across a wide range of temperatures, when compared to their polydisperse counterparts. Although the formation of serum protein corona seems to be mainly determined by polymer shell composition—that is nearly constant by comparing monodisperse and polydisperse POEGMA shells—binding to APAs was favored by the presence of longer OEG segments. They are present within polydisperse POEG_*p*_MA shells and function as epitopes for significant antibody recognition.

These findings shine light on the principles required for designing PEG-based polymer shells for NPs, highlighting how a fine control over polymer architecture and its structural heterogeneity can be leveraged for generating nonimmunogenic formulations.

More generally, manipulation of polymer dispersity becomes an additional tool—besides composition and polymer architecture—for modulating the properties of macromolecular nanomaterials and their integration and functioning within biological media.

## Methods

### Materials

Oligo­(ethylene glycol) methyl ether methacrylate (OEGMA, molar mass *M*_*n*_ ∼ 500 Da, containing 100 ppm MEHQ and 200 ppm BHT as inhibitors, Sigma-Aldrich) was purified by filtration on a basic alumina column to remove inhibitors prior to polymerization. (±)-α-Lipoic acid/DL-thioctic acid (98%, abcr GmbH & Co. KG), 4-dimethylaminopyridine (DMAP, abcr GmbH & Co. KG) and tris­(2-pyridylmethyl) amine (TPMA, 98%) were purchased from abcr GmbH & Co. KG, and used as received. 2-Hydroxyethyl 2-bromoisobutyrate (HEBIB), tin­(II) 2-ethylhexanoate (Sn­(EH)_2_), copper­(II) bromide (CuBr_2_, 99%), 4-(2-hydroxyethyl)-1-piperazineethanesulfonic acid (HEPES), phosphate-buffered saline (PBS), bovine serum albumin (BSA), sodium citrate tribasic, and chloroauric acid were purchased from Sigma-Aldrich and used as received. 1-Ethyl-3-(3-dimethylaminopropyl) carbodiimide (EDC) was purchased from Fluorochem. Anti-PEG antibodies ab51257 (PEG-B-47, monoclonal) and SPR chips were purchased from abcam and Cytiva, respectively. Methanol (MeOH, >99.9%), acetonitrile (ACN), ethanol (EtOH), ethyl acetate (EtOAc), N,N-dimethylformamide (DMF, >99%) and deuterated chloroform (CDCl_3_) were purchased from Sigma-Aldrich and used as received.

### Isolation of Discrete OEG_8_MA

Commercial OEG_*p*_MA (*M*_*n*_ ∼ 500 Da) was dissolved in the eluent (98:2 EtOAc:MeOH) and subjected to flash chromatography using silica gel (Macherey-Nagel 60, 230–400 mesh granulometry (0.063–0.040 mm) under nitrogen (N_2_) pressure to isolate discrete OEG_*8*_MA. Single-fraction detection was performed using SiO_2_-coated TLC sheets (Macherey-Nagel PolygramSIL G/UV254, silica thickness 0.2 mm, Duren, Germany) stained with KMnO_4_ solution and the above-mentioned mobile phase. Solutions of OEG_*8*_MA were dried in a rotavapor under reduced pressure after the addition of hydroquinone, which acts as a radical inhibitor. Both the commercial macromonomer mixture and discrete OEG_*8*_MA were characterized by ^1^H NMR (Figure S1) and ultra performance liquid chromatography, UPLC (Figure 1), and they were stored at −20 °C.

### UPLC

The purity of the compounds was determined by UPLC performed with an Agilent 1290 Infinity LC System (Agilent Technologies, Milan, Italy), equipped with a binary pump and a diode array detector (190–500 nm) set at 220 nm and ZORBAX Eclipse XDB-C18 column (2.1 mm × 50 mm, 1.8 μm). The eluent was based on a gradient from 95:5 H_2_O:ACN (both with 0.1% TFA) to 100% ACN over a 25 min run.

### Electrospray Ionization/Mass Spectrometry-High Performance Liquid Chromatography (ESI/MS-HPLC)

Low resolution mass spectra of OEG_*8*_MA and OEG_*p*_MA were obtained with either a 1100 Series Agilent Technologies system, equipped with a binary pump (G1312A) and MSD SL trap mass spectrometer (G2445D SL) or with a Waters Micromass Q-ToF micro mass spectrometer. High resolution mass analysis of macromonomers was performed with a Xevo G2-XS Q-ToF. In all cases the instruments were equipped with an ESI source, either in positive or negative mode. Sample solutions were prepared in ACN to achieve a concentration within the range 10^–3^–10^–4^ M.

### Synthesis of Disulfide Containing ATRP Initiator

(±)-α-Lipoic acid (248 mg, 1.2 mmol, 1.2 equiv) was dissolved in 5 mL of anhydrous DCM under argon. Then, HEBIB (211 mg, 145 μL, 1 mmol, 1 equiv) was added and the solution was stirred for some minutes (the solution was wrapped in aluminum foil due to the sensitivity of lipoic acid toward light). A suspension of EDC (288 mg, 1.5 mmol, 1.5 equiv) and DMAP (36.7 mg, 300 μmol, 0.3 equiv) in DCM was added to the solution at 0 °C and stirred for 15 min. Later, the final solution was left stirring for 24 h at room temperature under Ar, purified by flash chromatography and dried under reduced pressure. (Yield: 76%, 304 mg, 762 μmol). The disulfide containing ATRP initiator was characterized by ^1^H NMR spectroscopy (Figure S3).

### Synthesis of POEG_p_MA and POEG_*8*_MA for Functionalization of Au NPs

POEG_*p*_MA and POEG_*8*_MA were synthesized by ARGET ATRP using 1 M OEG_*p*_MA or OEG_*8*_MA and a polymerization mixture comprising—OEGMA:initiator:CuBr_2_:TPMA:Sn­(EH)_2_ with relative concentrations 50:1:0.05:0.07:0.05 in DMF. For the synthesis of POEG_*p*_MA, OEG_*p*_MA (2.5 g, 5 mmol), initiator (0.1 mL, 0.1 mmol), CuBr_2_ (1.12 mg, 5 × 10^–3^ mmol), TPMA (2.03 mg, 7 × 10^–3^ mmol), and DMF (1.4 mL) were poured into a 15 mL Schleck flask and degassed with Ar for 30 min. Then, in a 25 mL round-bottom flask, Sn­(EH)_2_ (2.03 mg, 5 × 10^–3^ mmol) was dissolved in anisole and degassed with Ar for 30 min. Finally, 0.5 mL of Sn­(EH)_2_ solution was added to the solution containing the monomer. The polymerization was carried out at room temperature for 12 h and stopped by exposure to air. The final product was obtained after purification by dialysis (cut off 1 kDa) in MeOH for 2 days, followed by solvent evaporation under reduced pressure. A similar polymerization and purification procedure was followed for the synthesis of structurally monodisperse POEG_*8*_MA.

### Synthesis of Citrate-Stabilized Au NPs

A solution of 45 mL of sodium citrate tribasic 38.8 mM in H_2_O was added in one shot to a 0.4 L boiling solution of 1 mM HAuCl_4_. The resulting mixture was heated at 130 °C under stirring for 15 min and, later, cooled down to room temperature. Finally, the mixture was filtered using a Corning 500 mL bottle top filter.

### Functionalization of Au NPs with POEG_p_MA and POEG_*8*_MA Brushes

Au NP dispersions were concentrated using 50 mL Amicon ultrafiltration tubes containing a cellulose membrane with a cutoff of 100 kDa to reach a concentration of 100 mM. To the obtained dispersion 0.4 mL of a 0.5 mM polymer solution in EtOH were added. The mixture was wrapped with an aluminum foil and left stirring for 4 days. Following functionalization with POEG_*p*_MA or POEG_*8*_MA adsorbates core-polymer shell NPs were purified from the excess of unbound polymer by centrifugation (20,000 rcf, 45 min, 15 °C, ×2) while removing the supernatant and using Amicon ultrafiltration (1000 rcf, 10 min, 25 °C, ×3).

### NP Stability Test

NP stability tests were carried out in a 96-well plate, with a total volume of 200 μL per well. Aliquots of Au NPs colloidal dispersions (3.5 nM) and increasing volumes of NaCl_(aq)_ were mixed and left under gentle shaking for 30 min, after which they were characterized by UV–vis spectroscopy. The final NaCl concentrations used for the stability tests were 0.4, 0.8, 1.2, 1.6, and 2.0 M.

### NP Temperature-induced Aggregation Test

NPs samples were prepared at a concentration of 3.5 nM in a solution of NaCl 2 M in water using a temperature ramp from 37 to 65 °C with a heating/cooling rate of 1 °C min^–1^ to study aggregation of NPs (*D*_H_ vs *T*).

### Turbidity Test

Thermoresponsive behavior of POEGMA@NPs was analyzed by using DLS (transmittance vs *T*). Turbidity measurements were carried out in the temperature range 37–60 °C with heating/cooling rate of 1 °C min^–1^. Cloud point temperatures (*T*_cp_) were assigned at temperatures that showed a 50% decrease in transmittance with respect to the value recorded at 37 °C.

### Grafting-to Preparation of POEGMA Brushes on Au Substrates

Au-coated silicon wafers were cut into 1.5 cm × 1.5 cm pieces, cleaned using piranha solution (H_2_SO_4_:H_2_O_2_ 3:1 v/v) for 1 min, rinsed with ultrapure water and EtOH, and dried under a stream of N_2_. Freshly cleaned substrates were immersed for 1 h in 1 mg mL^–1^ of POEGMA solutions in ethanol. Following grafting-to assembly, the substrates were washed extensively with ethanol and dried under a stream of N_2_.

### Variable Angle Spectroscopy Ellipsometry (VASE) of POEGMA Brushes

Dry thickness (*T*_dry_) of POEGMA brushes was evaluated using M-2000F variable-angle spectroscopic ellipsometer from J. A. Woollam Co. (Lincoln, NE). The measurements were recorded within a range of wavelengths included between 400 and 850 nm using focusing lenses at 65° from the surface normal. Each data point resulted from an average of 20 measurements. In the case of POEGMA brushes grafted on Au, the VASE data were fitted using a bilayer model (Au and organic adlayer) using the analysis software WVASE32. The thickness of Au layer (200 nm) was assumed to be constant. The refractive index and extinction coefficient, *n* and *k* values respectively, for Au were fitted by measuring a freshly cleaned Au substrate. The organic adlayer (polymer brushes) was fitted using the Cauchy model approximated to *n* = *A* + *B*/λ^2^ (*A* = 1.45 and *B* = 0.01). Four different substrates were analyzed for each POEGMA film, and five points were measured on each sample to calculate the mean values of *T*_dry_ and their standard deviations.

### Atomic Force Microscopy (AFM)

Normal force measurements were performed by using MFP3D AFM (Asylum Research, Oxford Instruments, Santa Barbara). Colloidal AFM probes were prepared by affixing a silica colloid with a diameter of approximately 16 μm (EKA Chemicals AB, Kromasil R, Sweden) on a tipless cantilever (HQ:CSC38/Cr–Au, MikroMasch, Bulgaria) using a home-built micromanipulator. Symmetrical measurements (brush-vs-brush) tribopairs, the colloidal probes were coated with 3 nm of Cr followed by 20 nm of Au by using a thermal evaporator (MED020 coating system, BAL-TEC, Balzers, Lichtenstein). Subsequently, the gold coated colloidal probes were treated with UV-Ozone (Ossila Ltd., UK) for 30 min prior to the functionalization. The functionalization of the Au-coated colloidal probes was carried out by immersing them in ethanol solutions containing 1 mg mL^–1^ POEGMA adsorbates.

The normal (*K*_N_) spring constants for the cantilevers were obtained by Sader’s method before attaching the colloid, using the online calibration platform (https://ampc.ms.unimelb.edu.au/afm/calibration.html). The dimensions of the cantilever were taken from the manufacturer’s specifications. The obtained values of *K*_N_ are reported in Table S3.

The AFM measurements were performed in a liquid cell containing 1 mM HEPES buffer solution at pH = 7.4 on the Au substrates with POEGMA films grafted.

Adhesion tests were performed by using force-volume maps over a scan size of 20 μm (10 force points by 4 force lines) for every position analyzed. During the tests, the force distance, scanning rate and velocity were maintained at 1 μm, 0.99 Hz, and 1.98 μm s^–1^, respectively.

### BSA-NPs Interaction Test

NPs samples were prepared at a concentration of 3.5 nM and incubated in a solution of BSA 1 mg mL^–1^ for 30 min prior to measurement. DLS was used to record intensity profiles.

### Protein Corona Formation on Core–Shell NPs

The formation of protein corona on citrate@AuNPs, POEG_*p*_MA@AuNPs, and POEG_8_MA@AuNPs was evaluated by incubating 2.5 nM dispersions with 60% (v/v) HS/40% PBS for 20 min at 37 °C in a total volume of 300 μL. After this time, cold PBS (pH = 7.4) 10 mM was added to reach 1 mL and NPs were recovered by spinning (90 min, 13,000 rpm at 4 °C). NPs were further washed by centrifugation (2 × 90 min, 13,000 rpm at 4 °C) with concomitant removal of the supernatant.

### Sodium Dodecyl Sulfate-Polyacrylamide Gel Electrophoresis (SDS-PAGE) and Silver Staining

After the centrifugation steps, core–shell NPs were dissolved in 25 μL of loading sample buffer (62.5 mM Tris-HCl, pH 6.8, 2% w/v SDS, 25% v/v glycerol, 0.01% w/v bromophenol blue, with β-mercaptoethanol). After being heated at 95 °C for 5 min, equal volumes (12 μL) of samples were subjected to SDS-PAGE in 12% acrylamide gels. NP-independent protein recovery was assessed by running mock samples in protein media. Silver staining was used to reveal separated proteins. Gels were fixed for 30 min in 50% v/v methanol 10% v/v acetic acid and then incubated for 15 min in 5% v/v methanol, 1% v/v acetic acid, washed three times with water, and exposed for 90 s to thiosulfate solution (200 μg mL^–1^ Na_2_S_2_O_3_ pentahydrate). After extensive washing with water, gels were incubated in the dark for 30 min with 0.2 g L^–1^ AgNO_3_, rinsed, and developed for 5–15 min with a solution containing 60 mg mL^–1^ Na_2_CO_3_, 4 μg mL^–1^ Na_2_S_2_O_3_ pentahydrate, and 0.01875% v/v formaldehyde. The reaction was stopped by the addition of 6% v/v acetic acid and the gel was dried. Finally, densitometric analysis of the corresponding SDS-PAGE bands was performed by using ImageJ software by taking every lane separately to measure the total amount of proteins.

### Nuclear Magnetic Resonance Spectroscopy

^1^H NMR spectra were recorded using Bruker Advanced III 500 MHz spectrometer at room temperature and using CDCl_3_ as solvent. Chemical shifts (δ) were given in ppm relative to the signal of the solvent.

### Size Exclusion Chromatography (SEC)

SEC was performed using an Agilent 1260 Infinity gel permeation chromatography equipped with a refractive index (RI) detector and two PL gel Mixed-D columns (300 mm, 5 μm) connected in series to determine the number average molecular weight (*M*_n_) and dispersity (*Đ*) of polymers. The column compartment and RI detector were at 70 and 50 °C, respectively, and the eluent used was DMF containing 10 mM LiBr at a flow rate of 1 mL min^–1^. Every sample (polymer concentration ∼ 2 mg mL^–1^) was prepared by filtering through neutral alumina over a PTFE membrane with a porosity of 0.20 μm. SEC was calibrated with 12 linear poly­(methyl methacrylate) standards (*M*_n_ = 540–2.21 × 10^6^ Da).

### Dynamic Light Scattering (DLS)

The hydrodynamic particle size, Z-potential, and ramping experiments of core–shell NPs were measured with a Litesizer DLS 500 instrument from Anton Paar. Instead, the BSA-NP interaction test was performed using a Malvern Zetasizer Nano-S equipped with a HeNe laser (633 nm) and a Peltier 124 thermostatic system. Measurements were performed at 25 °C in water or PBS buffer (pH = 7.4).

### Transmission Electron Microscopy (TEM)

Images were recorded on a FEI Tecnai G12 microscope operating at 100 kV and acquired with an OSIS Veleta 4 K camera. ImageJ was used to measure the diameter of >900 nanoparticles and to calculate average diameter and area for grafting density estimation.

### Thermogravimetric Analysis (TGA)

TGA was performed with a TA Q5000 IR instrument (TA Waters), in a constant airflow with a heating rate of 10 °C min^–1^. 100 μL aliquots of NP suspension were weighed on alumina cups and heated from 30 to 1000 °C. In order to determine the amount of polymer bound to the NPs surface and calculate the grafting density, only the mass loss recorded between 100 and 700 °C was considered.

The grafting density was calculated using eq 1, where the moles of ligand (*n*_ligand_) were determined from the mass loss detected by TGA and the ligand molecular weight obtained by SEC, *N*_A_ is the Avogadro number, the total number of NPs (*N*_NPs_) was calculated from the TGA inorganic residue and the mass of a single nanoparticle (ρ = 19.32 g cm^–3^) and the surface area of a single nanoparticle (*A*_NP_) was obtained from TEM data.

1 $$\sigma =\frac{\mathrm{ligand molecules grafted onto NPs}}{\mathrm{total surface area of NPs}}=\frac{n_{\mathrm{ligand}}\cdot N_{A}}{N_{\mathrm{NPs}}\cdot A_{\mathrm{NP}}}$$

### UV–Vis Spectroscopy

UV–vis absorption spectra were recorded in water or PBS on a Varian Cary 50 spectrophotometer with 1 cm path length quartz cuvettes. The spectrophotometer was equipped with thermostated cell holders. For the salt aggregation test, TECAN infinite200 PRO was used as a plate reader.

### Surface Plasmon Resonance (SPR)

SPR analyses were performed on a GE/Cytiva Biacore X-100 dual flow-cell instrument. On a bare gold SPR chip, POEGMA brushes were immobilized *in situ* (flow rate 5 μL/min, contact time 1000 s) on the cell 2 (active cell) yielding a final immobilization level of 1420.5 response units (RU) and 1548.5 RU for POEG_*p*_MA and POEG_8_MA, respectively. The chip was challenged with increasing concentrations of APAs (flow rate 30 μL min^–1^; contact time 540 s; dissociation time 900 s). All the measurements were carried out at 25 °C in HEPES buffer (10 mM HEPES, pH 7.4). Between two consecutive runs, an injection of 0.15 μM BSA solution was performed to avoid nonspecific binding of APAs on the gold chip (contact time 180 s; dissociation time 60 s) and the regeneration was achieved with a solution of NaOH 0.2 M (contact time 30 s; stabilization time 30 s).

Each sensorgram was corrected for the corresponding baseline, obtained on the reference flow cell. The binding data was analyzed using the BIAevaluation software.

## Supplementary Material



## Notes

ESI/MS data; ^1^H NMR spectra; TEM analysis; DLS data; TGA analysis; additional UV–vis spectra; surface fluoroimmunoassay tests (PDF)

## References

[1] Pavón C., Benetti E. M., Lorandi F. (2024). Polymer Brushes on Nanoparticles
for Controlling the Interaction with Protein-Rich Physiological Media. Langmuir.

[2] Boyer C., Whittaker M. R., Bulmus V., Liu J. Q., Davis T. P. (2010). The design
and utility of polymer-stabilized iron-oxide nanoparticles for nanomedicine
applications. NPG Asia Mater..

[3] Li D. Y., Xu L. Z., Wang J., Gautrot J. E. (2021). Responsive Polymer
Brush Design and Emerging Applications for Nanotheranostics. Adv. Healthcare Mater..

[4] Krishnamoorthy M., Hakobyan S., Ramstedt M., Gautrot J. E. (2014). Surface-Initiated
Polymer Brushes in the Biomedical Field: Applications in Membrane
Science, Biosensing, Cell Culture, Regenerative Medicine and Antibacterial
Coatings. Chem. Rev..

[5] Walkey C. D., Chan W. C. W. (2012). Understanding
and controlling the interaction of nanomaterials
with proteins in a physiological environment. Chem. Soc. Rev..

[6] Shi D., Beasock D., Fessler A., Szebeni J., Ljubimova J. Y., Afonin K. A., Dobrovolskaia M. A. (2022). To PEGylate
or not to PEGylate: Immunological
properties of nanomedicine’s most popular component, polyethylene
glycol and its alternatives. Adv. Drug Delivery
Rev..

[7] Chen B. M., Cheng T. L., Roffler S. R. (2021). Polyethylene
Glycol Immunogenicity:
Theoretical, Clinical, and Practical Aspects of Anti-Polyethylene
Glycol Antibodies. ACS Nano.

[8] Moreno A., Pitoc G. A., Ganson N. J., Layzer J. M., Hershfield M. S., Tarantal A. F., Sullenger B. A. (2019). Anti-PEG
Antibodies Inhibit the Anticoagulant
Activity of PEGylated Aptamers. Cell Chem. Biol..

[9] Ibrahim M., Ramadan E., Elsadek N. E., Emam S. E., Shimizu T., Ando H., Ishima Y., Elgarhy O. H., Sarhan H. A., Hussein A. K., Ishida T. (2022). Polyethylene glycol (PEG): The nature,
immunogenicity, and role in the hypersensitivity of PEGylated products. J. Controlled Release.

[10] Bigini P., Gobbi M., Bonati M., Clavenna A., Zucchetti M., Garattini S., Pasut G. (2021). The role and impact
of polyethylene
glycol on anaphylactic reactions to COVID-19 nano-vaccines. Nat. Nanotechnol..

[11] Ju Y., Carreno J. M., Simon V., Dawson K., Krammer F., Kent S. J. (2023). Impact of anti-PEG
antibodies induced by SARS-CoV-2
mRNA vaccines. Nat. Rev. Immunol..

[12] Ju Y., Lee W. S., Pilkington E. H., Kelly H. G., Li S. Y., Selva K. J., Wragg K. M., Subbarao K., Nguyen T. H. O., Rowntree L. C. (2022). Anti-PEG
Antibodies Boosted in Humans
by SARS-CoV-2 Lipid Nanoparticle mRNA Vaccine. ACS Nano.

[13] Hoogenboom R. (2022). The future
of poly­(2-oxazoline)­s. Eur. Polym. J..

[14] Lorson T., Lübtow M. M., Wegener E., Haider M. S., Borova S., Nahm D., Jordan R., Sokolski-Papkov M., Kabanov A. V., Luxenhofer R. (2018). Poly­(2-oxazoline)­s
based biomaterials:
A comprehensive and critical update. Biomaterials.

[15] Bauer T. A., Simić L., Van Guyse J. F. R., Duro-Castaño A., Nebot V. J., Barz M. (2024). Polypept­(o)­ides
– Origins,
synthesis, applications and future directions. Prog. Polym. Sci..

[16] Rheinberger T., Rabaux O., Jérôme C., Wurm F. R. (2023). The future
of polyphosphoesters. Eur. Polym. J..

[17] Qi Y. Z., Simakova A., Ganson N. J., Li X. H., Luginbuhl K. M., Ozer I., Liu W. G., Hershfield M. S., Matyjaszewski K., Chilkoti A. (2017). A brush-polymer/exendin-4
conjugate
reduces blood glucose levels for up to five days and eliminates poly­(ethylene
glycol) antigenicity. Nat. Biomed. Eng..

[18] Ozer I., Pitoc G. A., Layzer J. M., Moreno A., Olson L. B., Layzer K. D., Hucknall A. M., Sullenger B. A., Chilkoti A. (2022). PEG-Like Brush Polymer Conjugate
of RNA Aptamer That
Shows Reversible Anticoagulant Activity and Minimal Immune Response. Adv. Mater..

[19] Ozer I., Kelly G., Gu R. P., Li X. H., Zakharov N., Sirohi P., Nair S. K., Collier J. H., Hershfield M. S., Hucknall A. M., Chilkoti A. (2022). Polyethylene
Glycol-Like Brush Polymer
Conjugate of a Protein Drug Does Not Induce an Antipolymer Immune
Response and Has Enhanced Pharmacokinetics than Its Polyethylene Glycol
Counterpart. Adv. Sci..

[20] Joh D. Y., Zimmers Z., Avlani M., Heggestad J. T., Aydin H. B., Ganson N., Kumar S., Fontes C. M., Achar R. K., Hershfield M. S. (2019). Architectural Modification
of Conformal PEG-Bottlebrush Coatings Minimizes Anti-PEG Antigenicity
While Preserving Stealth Properties. Adv. Healthcare
Mater..

[21] Hu F. X., Neoh K. G., Cen L., Kang E. T. (2006). Cellular response
to magnetic nanoparticles ″PEGylated″ via surface-initiated
atom transfer radical polymerization. Biomacromolecules.

[22] Chen J. F., Rizvi A., Patterson J. P., Hawker C. J. (2022). Discrete Libraries
of Amphiphilic Poly­(ethylene glycol) Graft Copolymers: Synthesis,
Assembly, and Bioactivity. J. Am. Chem. Soc..

[23] Fatimah, Gurnani P., Williams G. R. (2024). Investigating
oligo­(ethylene glycol) methacrylate thermoresponsive copolymers. Eur. Polym. J..

[24] Choi H., Schulte A., Müller M., Park M., Jo S. W., Schönherr H. (2021). Drug Release
from Thermo-Responsive Polymer Brush Coatings
to Control Bacterial Colonization and Biofilm Growth on Titanium Implants. Adv. Healthcare Mater..

[25] Müller S., Cavallaro A., Vasilev K., Voelcker N. H., Schönherr H. (2016). Temperature-Controlled
Antimicrobial Release from Poly­(diethylene glycol methylether methacrylate)-Functionalized
Bottleneck-Structured Porous Silicon for the Inhibition of Bacterial
Growth. Macromol. Chem. Phys..

[26] Yamamoto S.-i., Pietrasik J., Matyjaszewski K. (2007). ATRP synthesis of thermally responsive
molecular brushes from oligo­(ethylene oxide) methacrylates. Macromolecules.

[27] Wassel E., Jiang S. Y., Song Q. M., Vogt S., Nöll G., Druzhinin S. I., Schönherr H. (2016). Thickness Dependence of Bovine Serum
Albumin Adsorption on Thin Thermoresponsive Poly­(diethylene glycol)
Methyl Ether Methacrylate Brushes by Surface Plasmon Resonance Measurements. Langmuir.

[28] Jiang S. Y., Müller M., Schönherr H. (2019). Toward Label-Free Selective Cell
Separation of Different Eukaryotic Cell Lines Using Thermoresponsive
Homopolymer Layers. ACS Appl. Bio. Mater..

[29] Lee H., Lee E., Kim D. K., Jang N. K., Jeong Y. Y., Jon S. (2006). Antibiofouling
polymer-coated superparamagnetic iron oxide nanoparticles as potential
magnetic resonance contrast agents for in vivo cancer imaging. J. Am. Chem. Soc..

[30] Ohno K., Mori C., Akashi T., Yoshida S., Tago Y., Tsujii Y., Tabata Y. (2013). Fabrication
of Contrast Agents for
Magnetic Resonance Imaging from Polymer-Brush-Afforded Iron Oxide
Magnetic Nanoparticles Prepared by Surface-Initiated Living Radical
Polymerization. Biomacromolecules.

[31] Pavón C., Ongaro A., Filipucci I., Ramakrishna S. N., Mattarei A., Isa L., Klok H. A., Lorandi F., Benetti E. M. (2024). The Structural Dispersity
of Oligoethylene Glycol-Containing
Polymer Brushes Determines Their Interfacial Properties. J. Am. Chem. Soc..

[32] Romio M., Grob B., Trachsel L., Mattarei A., Morgese G., Ramakrishna S. N., Niccolai F., Guazzelli E., Paradisi C., Martinelli E. (2021). Dispersity within Brushes
Plays a Major Role in Determining Their Interfacial Properties: The
Case of Oligoxazoline-Based Graft Polymers. J. Am. Chem. Soc..

[33] Ogbonna N. D., Dearman M., Cho C.-T., Bharti B., Peters A. J., Lawrence J. (2022). Topologically Precise
and Discrete Bottlebrush Polymers:
Synthesis, Characterization, and Structure–Property Relationships. JACS Au.

[34] Conrad J. C., Robertson M. L. (2023). Shaping the Structure and Response of Surface-Grafted
Polymer Brushes via the Molecular Weight Distribution. JACS Au.

[35] Ogbonna N. D., Guragain P., Mayandi V., Sadrinia C., Danrad R., Jois S., Lawrence J. (2025). Discrete Brush
Polymers Enhance F
MRI Performance through Architectural Precision. J. Am. Chem. Soc..

[36] Dearman M., Ogbonna N. D., Amofa C. A., Peters A. J., Lawrence J. (2022). Versatile
strategies to tailor the glass transition temperatures of bottlebrush
polymers. Polym. Chem..

[37] Pester C. W., Benetti E. M. (2022). Modulation of Polymer
Brush Properties by Tuning Dispersity. Adv.
Mater. Interfaces.

[38] Wu H. S., Shi Y. P., Lin T. C., Abdullah A., Bockstaller M. R., Matyjaszewski K. (2025). Accelerated
Self-Healing and Property Recovery in Brush
Particle Solids Featuring Brush Dispersity. ACS Macro Lett..

[39] Harrisson S. (2018). The downside
of dispersity: why the standard deviation is a better measure of dispersion
in precision polymerization. Polym. Chem..

[40] Jakubowski W., Matyjaszewski K. (2006). Activators
regenerated by electron transfer for atom-transfer
radical polymerization of (meth)­acrylates and related block copolymers. Angew. Chem., Int. Ed..

[41] Jakubowski W., Min K., Matyjaszewski K. (2006). Activators
regenerated by electron
transfer for atom transfer radical polymerization of styrene. Macromolecules.

[42] Weisbecker C. S., Merritt M. V., Whitesides G. M. (1996). Molecular
self-assembly of aliphatic
thiols on gold colloids. Langmuir.

[43] Lévy R., Thanh N. T. K., Doty R. C., Hussain I., Nichols R. J., Schiffrin D. J., Brust M., Fernig D. G. (2004). Rational and combinatorial
design of peptide capping Ligands for gold nanoparticles. J. Am. Chem. Soc..

[44] Kreibig U., Genzel L. (1985). Optical-Absorption
of Small Metallic Particles. Surf. Sci..

[45] Storhoff J. J., Lazarides A. A., Mucic R. C., Mirkin C. A., Letsinger R. L., Schatz G. C. (2000). What controls the optical properties of DNA-linked
gold nanoparticle assemblies?. J. Am. Chem.
Soc..

[46] Gillich T., Acikgöz C., Isa L., Schlüter A. D., Spencer N. D., Textor M. (2013). PEG-Stabilized Core–Shell
Nanoparticles: Impact of Linear versus Dendritic Polymer Shell Architecture
on Colloidal Properties and the Reversibility of Temperature-Induced
Aggregation. ACS Nano.

[47] Morgese G., Shaghasemi B. S., Causin V., Zenobi-Wong M., Ramakrishna S. N., Reimhult E., Benetti E. M. (2017). Next-Generation
Polymer Shells for Inorganic Nanoparticles are Highly Compact, Ultra-Dense,
and Long-Lasting Cyclic Brushes. Angew. Chem.,
Int. Ed..

[48] Schroffenegger M., Leitner N. S., Morgese G., Ramakrishna S. N., Willinger M., Benetti E. M., Reimhult E. (2020). Polymer Topology Determines
the Formation of Protein Corona on Core-Shell Nanoparticles. ACS Nano.

[49] Lutz J. F. (2008). Polymerization
of oligo­(ethylene glycol) (meth)­acrylates: Toward new generations
of smart biocompatible materials. J. Polym.
Sci., A Polym. Chem..

[50] Wang Y. Q., Tan X., Zhang Y. H., Hill D. J. T., Zhang A. F., Kong D. H., Hawker C. J., Whittaker A. K., Zhang C. (2024). Discrete Side Chains
for Direct Tuning Properties of Grafted Polymers. Macromolecules.

[51] Morbidelli M., Romio M., Chandorkar Y., Gogos A., Hirsch C., Kolrosova B., Trachsel L., Lorandi F., Badocco D., Pastore P. (2025). The Topology of Poly­(2-methyl-2-oxazine) Shells on
Nanoparticles Determines Their Interaction with Serum and Uptake by
Immune Cells. Biomacromolecules.

[52] Tavano R., Morillas-Becerril L., Geffner-Smith A., Ronzani G., Gervasutti R., Arrigoni G., Battisti I., Morbidelli M., de Laureto P. P., Palazzi L. (2025). Species differences
in opsonization and phagocyte recognition of preclinical poly-2-alkyl-2-oxazoline-coated
nanoparticles. Nat. Commun..

[53] Fedeli C., Segat D., Tavano R., Bubacco L., De Franceschi G., de Laureto P. P., Lubian E., Selvestrel F., Mancin F., Papini E. (2015). The functional
dissection of the
plasma corona of SiO-NPs spots histidine rich glycoprotein as a major
player able to hamper nanoparticle capture by macrophages. Nanoscale.

[54] Villela S. M. A., Kraïem H., Bouhaouala-Zahar B., Bideaux C., Lara C. A. A., Fillaudeau L. (2020). A protocol
for recombinant protein quantification by densitometry. MicrobiologyOpen.

[55] Zhang P., Sun F., Hung H. C., Jain P., Leger K. J., Jiang S. Y. (2017). Sensitive
and Quantitative Detection of Anti-Poly­(ethylene glycol) (PEG) Antibodies
by Methoxy-PEG-Coated Surface Plasmon Resonance Sensors. Anal. Chem..

[56] Nguyen M. T. T., Shih Y. C., Lin M. H., Roffler S. R., Hsiao C. Y., Cheng T. L., Lin W. W., Lin E. C., Jong Y. J., Chang C. Y., Su Y. C. (2022). Structural
determination of an antibody
that specifically recognizes polyethylene glycol with a terminal methoxy
group. Commun. Chem..

[57] Xiao Y. F., Lian X. Z., Sun Y. H., Sung Y. C., Vaidya A., Chen Z. X., Gupta A., Chatterjee S., Zheng L. N., Guerrero E. (2025). High-density
brush-shaped
polymer lipids reduce anti-PEG antibody binding for repeated administration
of mRNA therapeutics. Nat. Mater..

