# Supplementary material for: The Role of Structural Dispersity of Polymer Brushes in Determining the Colloidal Stability of Core–Shell Nanoparticles and Their Interaction with Anti-PEG Antibodies
Source: JACS Au. 2025 Aug 26;5(9):4519–29. doi: 10.1021/jacsau.5c00852 (PMC12458011; doi:10.1021/jacsau.5c00852)
Supplement: Supplementary file 1 [file au5c00852_si_001.pdf]

SUPPORTING INFORMATION

The Role of Structural Dispersity of Polymer Brushes in Determining the Colloidal Stability of Core-Shell Nanoparticles and Their Interaction with Anti-PEG Antibodies

Carlos Pavón,[1] Antonella Grigoletto,[2] Verena Kempkes,[3] Ander Eguskiza,[4] Maria Morbidelli,[5] Roberto Fiammengo,[4] Emanuele Papini,[5] Andrea Mattarei,[2] Gianfranco Pasut,[2] Krzysztof Matyjaszewski,[3]* Francesca Lorandi,[1]* Edmondo M. Benetti[1]**

[1] Laboratory for Macromolecular and Organic Chemistry, Department of Chemical Sciences, University of Padova, via Marzolo 1, 35131 Padova, Italy.

[2] Department of Pharmaceutical and Pharmacological Sciences, University of Padova, Via Marzolo 5, 35131 Padova, Italy

[3] Department of Chemistry, Carnegie Mellon University, 4400 Fifth Avenue, Pittsburgh, Pennsylvania 15213, United States.

[4] Department of Biotechnology, University of Verona, 37134 Verona, Italy.

[5] Department of Biomedical Sciences, University of Padova, 35121 Padova, Italy.

Table S1. OEG$_p$MA distributions with the abundance (% mol) of every peak taken from UPLC chromatograms.

Repeating units (n)	Number average molecular weight (M_n) [Da]	Retention time (RT) [min]	Concentration [% mol]
2	188	8.0	0.6
3	232	8.7	2.2
4	276	9.2	4.6
5	320	9.7	7.4
6	364	10.0	10.7
7	408	10.3	13.7
8	452	10.6	14.6
9	496	10.8	13.7
10	540	11.0	11.4
11	584	11.2	8.6
12	628	11.4	6.2
13	672	11.6	3.4
14	716	11.7	1.9
15	760	11.9	1.0

Table S2. OEG$_8$MA distributions with the abundance (% mol) of every peak taken from UPLC chromatograms.

Repeating units (n)	Number average molecular weight (M_n) [Da]	Retention time (RT) [min]	Concentration [% mol]
7	408	10.3	1.1
8	452	10.5	98.5
9	496	10.9	0.4

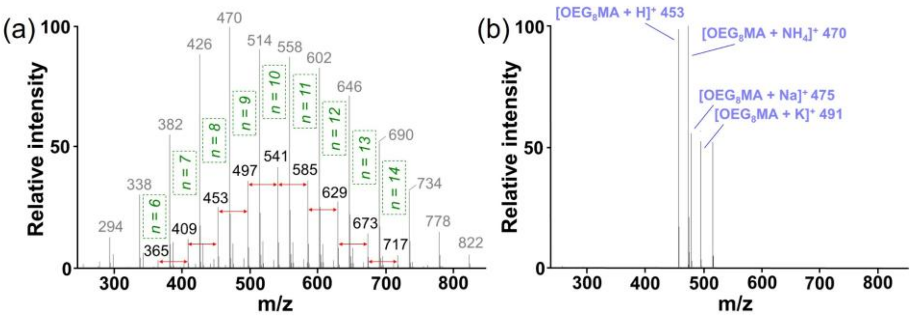

Figure S1. Electrospray ionisation/mass spectrometry (ESI/MS) spectrum of (a) OEG$_p$MA macromonomer mixture where m/z in black are referred to [OEG$_p$MA + H]$^+$ and in grey, [OEG$_p$MA + NH$_4$]$^+$. (d) ESI/MS spectrum of OEG$_8$MA where m/z = 453 for [OEG$_8$MA + H]$^+$, m/z = 470 for [OEG$_8$MA + NH$_4$]$^+$, m/z = 475 for [OEG$_8$MA + Na]$^+$, and m/z = 491 for [OEG$_8$MA + K]$^+$.

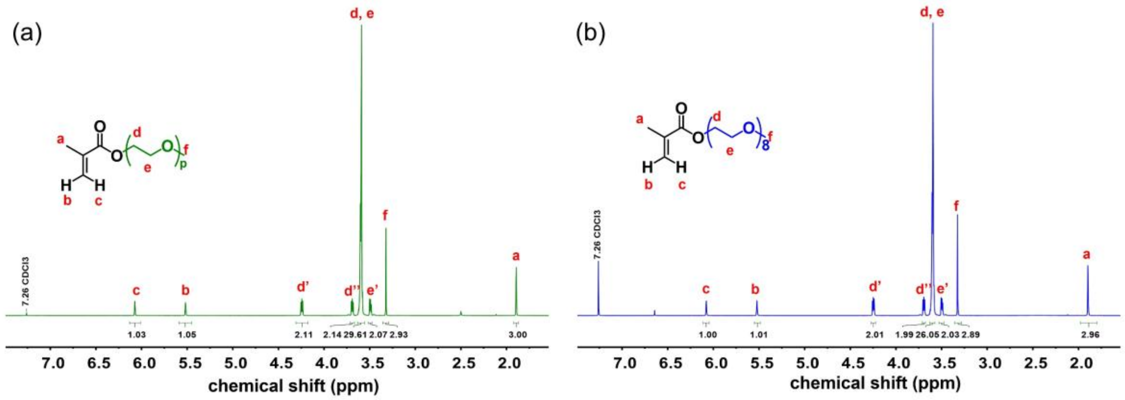

Figure S1. ^{1}H-NMR (500 MHz) spectra of macromonomers (a) OEG$_{p}$MA and (b) OEG$_{8}$MA recorded in $CDCl_3$.

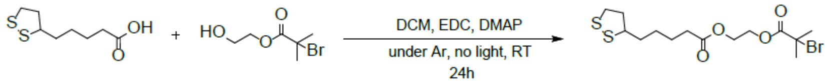

Figure S2. Synthesis of the initiator containing disulfide used for ATRP.

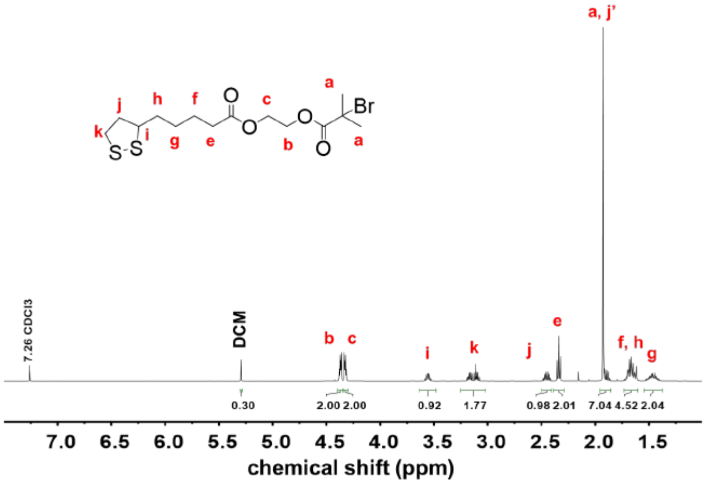

Figure S3. ^{1}H-NMR (400 MHz) spectrum of initiator containing disulfide recorded in $CDCl_3$.

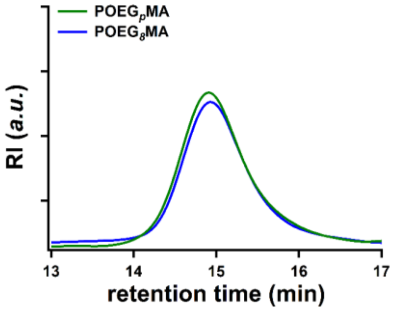

Figure S4. SEC elugrams of POEG$_{p}$MA and POEG$_{8}$MA recorded using DMF with 10 mM LiBr as eluent.

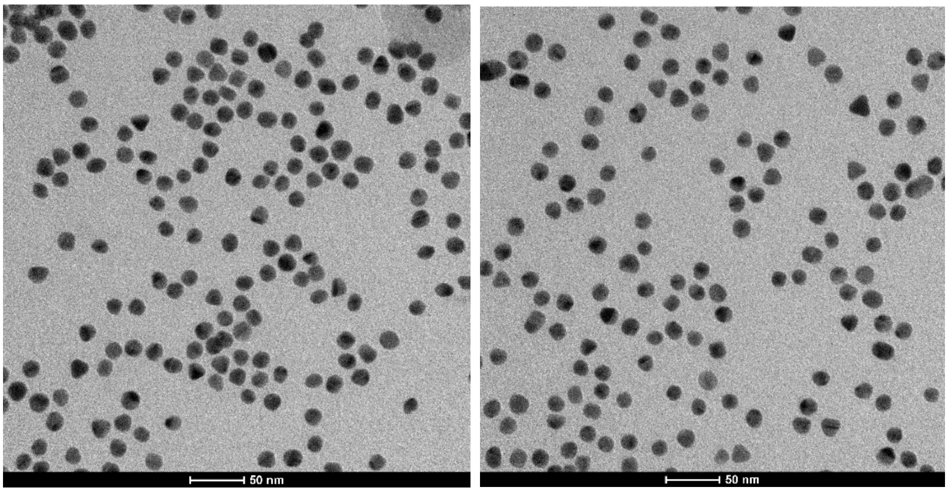

Figure S5. TEM images of POEG$_p$MA@AuNPs (on the left) and POEG$_8$MA@AuNPs (on the right).

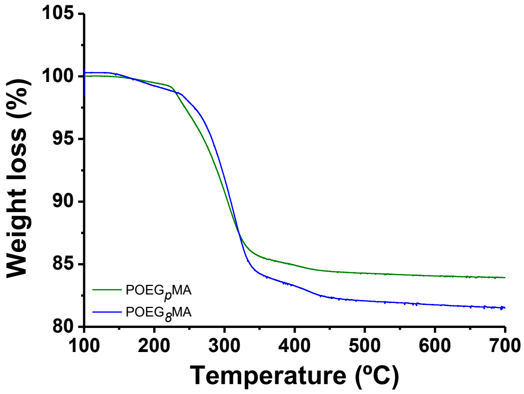

Figure S6. Representative TGA curve measured on the different batches of POEGMA-stabilized NPs in air (heating rate 10 °C/min, temperature range 100-700 °C).

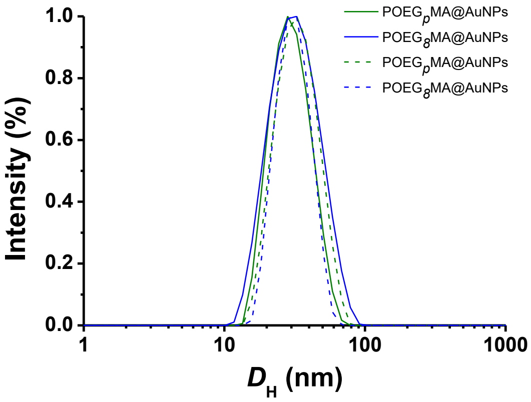

Figure S7. DLS profiles displaying normalized intensity-weighted D_H of 3.5 nM dispersions of POEG$_p$MA@AuNPs and POEG$_8$MA@AuNPs in PBS at t=0 and after one week incubation at room temperature (dashed lines).

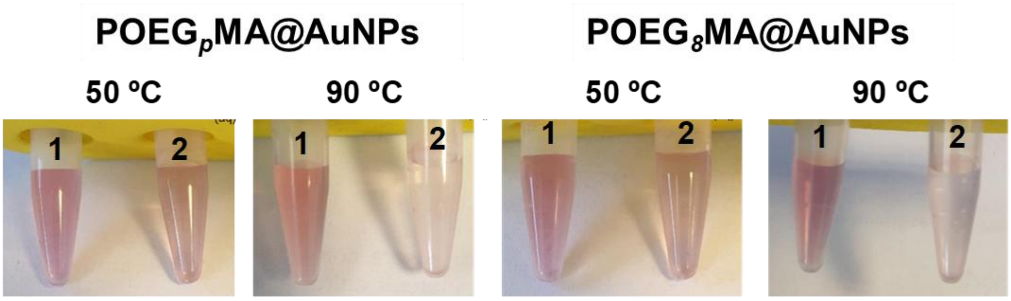

Figure S8. Comparison of the Au NPs colloidal solutions when applying a temperature ramp without (1) and with (2) 2 M NaCl $_{(aq)}$.

Table S3. Normal (K_N) spring constant of the probes used for the AFM measurements.

Cantilever	K_N [N m^{-1}]
brush-*vs*-brush	
POEG$_p$MA	0.180
POEG$_8$MA	0.162

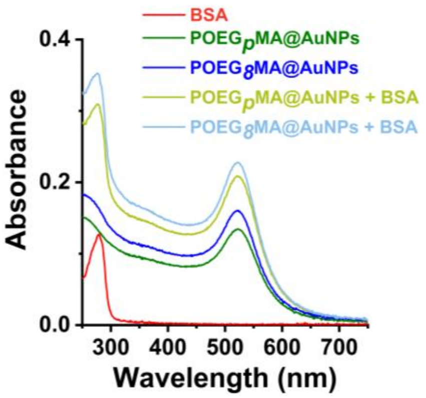

Figure S9. UV-vis spectra of POEG$_p$MA@AuNPs and POEG$_8$MA@AuNPs before and after incubation with BSA.

APA binding on POEGMA brushes by fluoroimmunoassay

In addition to SPR analysis, we have assessed the reactivity of POEG$_p$MA and POEG$_8$MA brushes toward a rabbit-derived monoclonal anti-PEG antibody using a surface fluoroimmunoassay, also comparing POEG$_3$MA, which was recently highlighted as non-immunogenic (1). In particular, POEG$_3$MA, POEG$_8$MA, and POEG$_p$MA brushes were grafted from SiO_x substrates following an already established protocol (2) to yield surface-grafted assemblies with dry thicknesses (T_{dry}) of 55.5 ± 3.9, 51.8 ± 3.7 nm, and

53.9 ± 2.6 nm, as measured by variable angle spectroscopic ellipsometer (VASE), for POEG$_3$MA, POEG$_8$MA, and POEG$_p$MA, respectively. Each polymer assembly was first incubated for 1 hour with 2 µg mL^{-1} APA which was applied directly to the surface and then rinsed with PBS. Surfaces were then incubated with 2 µg mL^{-1} solution of a secondary antibody donkey anti-rabbit IgG (Alexa Fluor® 488) for 30 min, rinsed with PBS and dried, carefully, with N_2. Fluorescence intensities were quantified by a photoluminescence spectrometer with a maximum excitation of 495 nm and emission of 519 nm. Compared to POEG$_p$MA, a significant decrease in fluorescence intensity was recorded for POEG$_8$MA confirming a much lower reactivity towards anti-PEG antibodies for brushes with homogeneous structure.

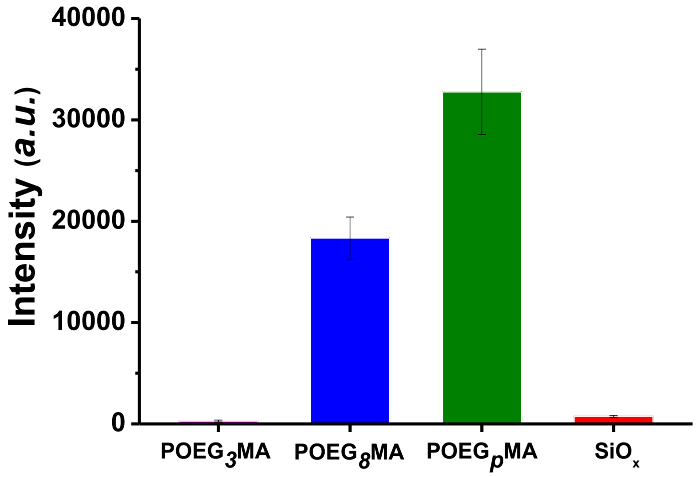

Figure S10. Fluorescence intensity recorded by photoluminescence spectroscopy on POEG$_3$MA, POEG$_8$MA, and POEG$_p$MA brushes first incubated for 1 hour with 2 µg mL^{-1} APA and subsequently subjected to 2 µg mL^{-1} solution of a secondary antibody donkey anti-rabbit IgG (Alexa Fluor® 488). The reported values are plotted as mean for two substrates and three different spots. The control was a bare SiO_x substrate.

References

(1) Ozer, I.; Kelly, G.; Gu, R. P.; Li, X. H.; Zakharov, N.; Sirohi, P.; Nair, S. K.; Collier, J. H.; Hershfield, M. S.; Hucknall, A. M.; et al. Polyethylene Glycol-Like Brush Polymer Conjugate of a Protein Drug Does Not Induce an Antipolymer Immune Response and Has Enhanced Pharmacokinetics than Its Polyethylene Glycol Counterpart. *Adv. Sci.* **2022**, *9* (11). DOI: ARTN 2103672 10.1002/advs.202103672.

(2) Pavón, C.; Ongaro, A.; Filipucci, I.; Ramakrishna, S. N.; Mattarei, A.; Isa, L.; Klok, H. A.; Lorandi, F.; Benetti, E. M. The Structural Dispersity of Oligoethylene

Glycol-Containing Polymer Brushes Determines Their Interfacial Properties. *J. Am. Chem. Soc.* **2024**, *146* (24), 16912-16919. DOI: 10.1021/jacs.4c05565.
